# Transcranial Magnetic Stimulation in Tourette Syndrome: A Historical Perspective, Its Current Use and the Influence of Comorbidities in Treatment Response

**DOI:** 10.3390/brainsci8070129

**Published:** 2018-07-06

**Authors:** Marco Grados, Rachel Huselid, Laura Duque-Serrano

**Affiliations:** 1 Johns Hopkins University School of Medicine, Baltimore, MD 21287, USA; 2 Johns Hopkins University Krieger School of Arts & Sciences, Baltimore, MD 21205, USA; rhuseli1@jhu.edu; 3 School of Medicine, Universidad de los Andes, Bogotá 111711, Colombia; l.duque75@uniandes.edu.co

**Keywords:** transcranial magnetic stimulation, tourette syndrome, insula, supplementary motor area

## Abstract

Background. Tourette syndrome (TS) is a childhood-onset neuropsychiatric disorder consisting of impairing motor and vocal tics which often persists adolescent and adult years. In this older refractory group, standard treatments such as pharmacotherapy and psychotherapeutic interventions may only have limited effects. Based on electrical cortical dysregulation in individuals with TS, a novel approach has employed brain stimulation strategies to modulate the putative aberrant neural electrical activity in pathways that may underlie tics, such as insula-supplementary motor area (SMA) connectivity. Methods. This review will examine all published clinical trials employing transcranial magnetic stimulation (TMS) to ameliorate tics, and discuss a framework for the pathophysiology of TS in relation to electrical brain activity. A framework for future research in tic disorders using TMS and imaging targeting neuroplasticity will be discussed. Results. Therapeutic electrical brain activity modulation with TMS has been carried out in stroke neuro-rehabilitation and neuropsychiatry, including trials in TS. Eleven trials document the use of TMS in TS targeting several brain areas, a positive effect is seen for those trials targeting the SMA. In particular, it appears that younger individuals with concurrent attention-deficit hyperactivity disorder (ADHD) benefit the most. Conclusions. TMS can be used as an effective tool to explore the psychophysiology of TS and potentially provide a therapeutic option. Ultimately, translational research using TMS in TS needs to explore connectivity differences pre- and post-treatment in individuals with TS that are linked to improvement in tic symptoms, with an emphasis on approaches using functional neuroimaging as well as other probes of neuroplasticity.

## 1. Introduction

Tourette syndrome (TS) is a developmental neuropsychiatric disorder characterized by a childhood onset, male predominance [1], familial underpinnings [2], and often a life-long course [3] Clinically, TS is defined by the presence of clinically impairing multiple motor, and at least one vocal, tic occurring for over 12 months [4]. TS is frequently comorbid with attention-deficit hyperactivity disorder (ADHD) and obsessive-compulsive disorder (OCD); additionally, an excess of autism spectrum disorder (ASD) traits, learning difficulties, sensory integration disorder are also commonly reported [5]. Etiologically, sharing of genetic vulnerabilities for OCD [6], ADHD [7], disorders related to grooming (trichotillomania, skin picking), and mood dysregulation (bipolar disorder) [8] have been suggested, while more recent findings point to deficits in neurodevelopmental aspects of synaptic function [9].

The pathophysiology of TS is not fully understood, but increased dopamine release in the striatum (putamen) in conjunction with low serotonergic tone [10], and developmentally immature brain connectivity [11] are posited, among competing theories. Clinically, anteceding the execution of motor tics, patients often report a premonitory urge, an uncomfortable sensation in a muscle group, which can be alleviated by the tic behavior [12]. The premonitory urge is related to a heightened interoceptive awareness, the sensation of internal bodily states, as opposed to exteroception, the sensation from cutaneous mechanoreception and proprioception [13]. Supporting this theoretical framework, the insula, the brain’s interoceptive center, is observed to be overactive before tic behaviors [14]. Further associating interoceptive abnormalities in TS to the origin of tics, resting-state functional magnetic resonance imaging (fMRI) probes of tic phenomena, show that the dorsal anterior insula is hyperconnected to the supplementary motor area (SMA), a motor planning area [15]. Given the functional properties of insular cortex, such as the integration of sensory and emotional information, and processing of bodily sensations [16], it is plausible that methods that modulate insular activity may decrease internal tic urges and reinforce urge suppression activity.

Among the methods to induce or suppress targeted neural activity, inducing neuroplastic changes in cortical neurocircuitry, is transcranial magnetic stimulation (TMS). TMS is a noninvasive procedure which employs extracranial magnetic fields to create cortical electrical activity, which creates a stimulatory impulse to cortical neurons. Depending on TMS parameters, either induction, suppression or neural activity can be attained. In TS, natural targets for TMS have historically included motor and premotor cortex [17], but more recently, the SMA [18]. The aim of this narrative review is to examine the historical context of the use of TMS in TS, and to highlight published studies which use TMS to ameliorate TS symptoms both in adults and children. A framework for understanding predictive factors for the efficacy of TMS in TS is also offered.

## 2. Transcranial Magnetic Stimulation

TMS is based on the application of a magnetic field generated by pulsating electrical currents in a coil applied to cranial structures, which itself induces electrical activity intracranially in discrete brain regions. Magnetic fields used in TMS are based on the Biot–Savart law, discovered by Jean-Baptiste Biot and Felix Savart in 1820, which explains how electrical current generate magnetic fields [19]. and Faraday’s law which postulates that fluctuating magnetic fields induce a “wave of electricity” [20]. These mechanisms generate, electricity in neuronal spaces and depolarize columns of neurons [21]. Technical considerations coil shape, brain region landmarks, and field strength (1.5–2 tesla). In repetitive TMS (rTMS), the magnetic stimulators that produce the electrical current are able to cycle at high rates to produce high pulse frequencies. High pulse frequencies generate a high degree of synchrony firing in neurons given that the period of inhibition after a TMS pulse lasts about 100 ms. The effect of TMS is able to establish hemispheric dominance [22]. And affect learning [23]. More recent data support rTMS reduction of inhibitory neurotransmission to facilitate associative plasticity in hippocampal slice cultures [24]. Enhancing associative synaptic plasticity [25]. The first therapeutic use of TMS was foreshadowed by Barker in 1985, reporting on the “novel method” of contactless stimulation of the human motor cortex [26]. By 2014, a targeted review of TMS in psychiatric disorders noted more than 600 papers mentioned TMS in relation with psychiatric disorders [27].

## 3. Neurocircuitry and Neurotransmitters Glutamate/GABA Suggest Abnormal Excitatory Activity in Tourette Syndrome 

In the pathophysiology of TS, cortico-striatal-thalamo-cortical circuits (CSTC) connecting brain cortical regions to basal ganglia, are critical to its presumed pathophysiology [28]. These loops are active in parallel, but also harbor interconnections to support the interplay of the motor, emotional, and cognitive domains [29]. A direct excitatory pathway from striatal medium spiny neurons (MSN) to globus pallidus, and an indirect inhibitory pathway from the globus pallidus through the subthalamic nucleus [30] set the stage for the basal ganglia to function as a modulating center for planned and executed movements, inhibiting motor mechanisms which compete with planned movements [31]. At the neurotransmitter level, dopamine and glutamate—via the glutamatergic subthalamic nucleus STN in the indirect pathway—are the key neurotransmitters implicated. Single photon emission tomography (SPECT) and positron emission tomography (PET) studies, which tag dopamine ligands with radioactive traceable isotopes confirm increased density of striatal dopamine transporter density [32,33], increased 18F-fluoro-dopa uptake in the left caudate [34], greater dopamine release in putamen after amphetamine challenge [35], and increased 11C-dihydro-tetrabenazine binding in the right ventral striatum [36]. Mesocortical dopaminergic inputs from the ventral tegmental area, in turn, can alter glutamatergic pyramidal neuronal excitability both directly and indirectly; the indirect pathway using modulation from gamma-amino-butyric acide GABAergic interneurons [37]. In addition, a crucial role is played by glutamate in tonic/phasic ventral tegmental dopaminergic signaling via excitatory ventral subiculum hippocampal glutamate neurons [38]. A transgenic mouse has modeled chronic glutamate excitation of striatal motor output biased towards overactivity of the motoric direct pathway and inactivity of the inhibitory indirect pathway. In this model, a hyperglutamatergic CSTC “tic circuit” is proposed [39]. The only human post-mortem study assessing brain glutamate levels found reduced amounts in globus pallidus interna, globus pallidus externa, and substantia nigra pars reticulata [40]. More recently, a genomic study implicates astrocytic function and glutamate and glucose metabolism with TS [41], and a case control study showed that a gamma-amino-butyric acide (SLC1A3; also known as excitatory amino acid transporter 1 or EAAT-1) variant (E219D) resulted in increased uptake of glutamate by astrocytes [42]. Despite pathophysiological studies implicating glutamate in TS, clinical trials have not provided robust support for use of glutamate-modulators in this condition [43]. However, resting-state fMRI and magnetic resonance spectroscopy (MRS) studies have continued to provide support for ”immature connectivity” [11] and reductions in striatal concentrations of glutamine, glutamate + glutamine (Glx) in TS. Glutamine levels in the striatum were inversely associated with tic severity, while thalamic glutamate was inversely associated with tic urges [44]. In summary, dopamine and glutamate influences are widely recognized in TS, however, the counterbalance to glutamate transmission in the CNS, inhibitory GABA loads, may also play an influential role in the expression of TS.

While the abnormal glutamatergic drive theory in TS favors an excess of glutamate in the origin of tics, a lack of neuronal inhibitory capacity could equally account for tics through an excitation-inhibition (E/I) imbalance of the GABA-glutamate-glutamine cycle [44]. For TS, a developmental dysfunction of GABAergic striatal interneurons is a plausible component of an E/I imbalance. GABA neurons, in fact, temporally antecede glutamate neurons in development and act as the first excitatory neurons, with GABA stimulation being the sole excitatory transmitter early in fetal development [45]. While a GABA-driven hyperexcitable state is developmentally present early in life, over time, inhibitory neurocircuitry achieves maturity through the ”GABA switch” from excitatory to inhibitory functions. This process in GABA neuron development may be facilitated by oxytocin [46] and is evolutionarily preserved: early in *C. elegans* development GABA neurons produce muscle contraction through depolarization, while at the mid-larva1 stage the same neurons are hyperpolarizing and relax muscle [47]. If this switch is incomplete it can lead to epileptogenesis [48] and possibly other neurodevelopmental conditions [49]. In humans, The E → I switch for GABA neurons occurs postnatally at weeks 1–2 [50], with GABA load in neonates being lower than that of children [51]. The lack of developmental maturity in TS [11], specifically in basal ganglia interneurons, may be one factor predisposing children to tics. One post-mortem study has found that the tangential migration of GABA parvalbumin-positive interneurons may be abnormal in TS, with lower densities of interneurons in caudate-putamen (striate nuclei) and a higher proportion in the internal globus pallidus [52]. In sum, the delayed maturation of striatal GABAergic interneurons is an attractive emerging model for TS, a model which has been recently shown to cause dystonia, a related movement disorder [53].

The influence of cortical GABA in the clinical expression of TS has been examined, suggesting decreased cortical GABA in TS, although with developmental differences. In children 5–12 years with TS, one study used 1H magnetic resonance spectroscopy (MRS) at 7T and found no cortical GABA perturbations, but confirmed abnormal glutamate function in the premotor cortex [54]. In another study of 8–12-year-old children with TS and healthy controls, a reduced GABA concentration in primary sensorimotor cortex was detected using MRS [55]. At older ages, GABA imbalances may be more evident in TS. In 15 adolescents with TS, similarly using 1H MRS at ultra-high field (7 T), GABA was increased in SMA and was negatively correlated with cortical excitability [56]. In brain regions dependent on GABA interneurons for E/I homeostasis, inhibitory synaptic potentials coming from even single striatal interneurons can easily modulate the origin of action potentials in a large number of projection neurons [57], implying that maturational or other dysfunction affecting interneurons can readily dysregulate cortical or striatal neurocircuitry. However, complicating the clinical argument for an E/I imbalance in TS, compensatory mechanisms in individuals with TS may develop over time due to chronic tic suppression, resulting in an acquired ”tonic” inhibition state [58].

Cortical probes of GABA function with TMS include use of a paired-pulse paradigm with TMS to measure the GABA-mediated short-interval intracortical inhibition (SICI). In SICI, subthreshold stimulation, not sufficient to produce a corticospinal output, is applied as a condition pulse 1–5 msec before the test suprathreshold pulse. This paired stimulation significantly suppresses the amplitude of the motor evoked potential (MEP) of the second pulse [59]. Several studies have explored the association of SICI with TS. Interestingly, tic severity and ADHD hyperactivity-impulsivity scores in children and adults are correlated with less intracortical inhibition (lower SICI), while there is no relationship with obsessive-compulsive disorder (OCD) severity scores. Also, intracortical facilitation, the production of larger MEPs through more widely spaced TMS paired pulses that facilitate motor output, is not related to ADHD, tic or OCD clinical severity [60]. The relationship between lower SICI and ADHD symptoms is attenuated significantly in the presence of dopamine receptor blockers, possibly based on the regulation of cortical inhibition by D1 and D4 receptors involved in glutamate pathways. Speculatively, the same mechanisms that regulate subcortical inhibition, which when dysregulated could lead to tics, may be those that influence cortical inhibition [61].

## 4. Brain Electrical Activity and Tourette Syndrome

Having established that glutamatergic and GABAergic transmission may have a role in the development and maintenance of tics in TS, electrical activity in TS has been explored. An examination of surface electromyography recorded from the right abductor digiti minimi muscle was completed after TMS application to the left motor cortex. While motor threshold and peripheral motor excitability were normal in TS individuals, the cortical silent period was shortened and intracortical inhibition reduced. A subgroup analysis revealed that these differences were prominent in the presence of tics [62]. A more recent study used single-pulse TMS in conjunction with a manual Go/NoGo task to investigate alterations in corticospinal excitability ahead of volitional movements. In ten adolescents with TS, corticospinal excitability was significantly reduced in TS in the period immediately preceding a finger movement. TMS-induced motor evoked potentials were also abnormal only in the TS group, suggesting an inability to modulate motor cortical excitability prior to tics [63]. Motor evoked potentials have also been studied in relation to paired associated learning in TS, in order to explore neuroplasticity potential in the disorder. Paired associative stimulation in healthy controls produces long-term improvement, showing long-term potentiation due to neuroplasticity; however, individuals with TS did not show the improvement when tested nine months after paired associative stimulation. There was a statistical signal for the association of less synaptic plasticity with more severe tics [64]. Others studies have also shown aberrant motor cortical plasticity in TS using the paired associative stimulation paradigm [65].

While these studies establish that cortical electrical activity is abnormal in TS, the influence of OCD and ADHD on brain electrical activity needs to be elucidated. A study comparing cortical and brainstem plasticity in individuals with TS versus OCD alone, found that abnormal plasticity is consistently present in TS, but is normal in those individuals with OCD alone [66]. Along these lines, the main comorbid disorder with TS, ADHD has also been found to be associated with increased cortical excitability. In one study, motor thresholds, GABA-determined SICI, intracortical facilitation, and short latency afferent inhibition were measured with TMS in 18 TS only, 6TS + ADHD, and 5TS + OCD individuals, in comparison to 24 healthy subjects. In one notable finding in this report, individuals with TS + ADHD had more intracortical facilitation than controls, compared to individuals with uncomplicated TS and TS + OCD. It appears that ADHD cortical instability confers greater brain electrical imbalance in addition to that already present in TS [67]. These findings underscore the notion that neuropsychiatric disorders, such as ADHD and TS, with excess movement may involve increased glutamatergic excitatory output from thalamus to cortical centers, resulting in either excessive motor dyscontrol (hyperactivity/impulsivity) or tic behaviors (suppressible but difficult to contain). In another study which used TMS to measure motor cortex inhibition in 36 children and adults with TS, severity of ADHD symptoms, and motor tics were independently associated with SICI (r(2) = 0.50; F[2,27] = 13.7; *p* < 0.001), particularly in subjects not taking neuroleptics (r(2) = 0.68; F[2,17] = 17.8; *p* < 0.0001). The correlation with cortical disinhibition was mostly dependent on ADHD symptom severity (r = 0.53; *p* = 0.003) compared to tic severity (r = 0.42; *p* = 0.02). A continuum of electrical brain overactivity may thus exist in a gradient from TS + ADHD, TS + OCD to OCD only. Critically, the findings suggest a potential preference for using comorbid groups as targeted subpopulations in TMS [60].

## 5. Transcranial Magnetic Stimulation as a Measure of the Electrophysiology in Tourette Syndrome

As TMS can measure cortical brain electrical activity, several studies have examined these properties in TS and found specific abnormalities. Orth et al. (1992) measured motor thresholds, input-output (I/O) curves, SICI, and cortical silent periods (SP) with TMS in 20 untreated TS participants (12 uncomplicated, four with comorbid ADHD, 4with comorbid OCD) and 24 healthy subjects. Tics were rated with standard clinical scales and detailed video analysis. There was lower corticospinal excitability at rest in TS compared to controls, speculating that this may be an adaptive response to reduce release of unwanted movements [68]. In another study measuring voluntary motor drive (VMD), the presence of distal motor tics correlated with lower VMD compared to non-distal motor tics and controls [69]. More dramatically, electrical activity measured in cortical brain areas in TS suggests lack of maturation of neurocircuitry, as evidenced by increased resting motor thresholds and variability of motor evoked potential responses in children with TS compared to adults [70]. In addition, lack of neuroplastic neuronal reserves has also been postulated to underlie the lack of natural compensatory ability to suppress and eliminate tics seen in healthy individuals. In order to test the neuroplasticity potential with TMS, Jackson et al. (2013) measured cortical excitability, using TMS-induced MEPs, and found them to be significantly reduced in the TS group in the period immediately preceding a finger movement [71]. These changes in electrical overactivity and lack of neuroplasticity in TS suggest a combination of a glutamatergic imbalance [44], a GABA interneuron deficiency [72], and given recent genetic data, an intrinsic synaptic function abnormality [73]. The latter hypothesis may lead to further studies in neuroplasticity effects of TMS in TS, based on the the long-term potentiation (LTP)-like and long-term depression (LTD)-like effects of brain stimulation [74], as shown additionally by in vitro organotypic preparations, in which repetitive magnetic stimulation induces long-lasting strengthening of glutamatergic synapses and remodeling of dendritic spines [25].

## 6. Transcranial Magnetic Stimulation as a Therapeutic Tool in Tourette Syndrome

Clinical trials using both transcranial direct current stimulation (tDCS; the delivery of constant, low direct current delivered via electrodes on the cranium) and rTMS have explored the safety and efficacy of noninvasive brain stimulation as a treatment for children and adults with TS. While tDCS has not been studied extensively as a therapeutic option in TS, it has shown promise for its possible efficacy and confirmed safety [75]. Sparse data include a clinical case study using tDCS targeting the left motor cortex in two adult patients with TS, in which tDCS resulted in a significant reduction in tics compared to sham treatment [76].

The use of rTMS as a treatment for TS has been studied far more extensively. Several early studies examined the efficacy of 1Hz rTMS targeting the left motor cortex and left pre-motor cortex and found no significant reduction in TS tic symptoms overall [17,77]. Subsequently, Chae et al. tested rTMS efficacy targeting the left motor cortex and left prefrontal cortex, and found that while there was no significant reduction in tic severity overall, there was a significant reduction of tic severity in the TS/OCD comorbid subgroup [78], suggesting that the presence of comorbidities might influence results. The positive effect of TMS in comorbid TS was also identified by Bloch et al., who targeted the SMA using deep rTMS. In that study, a significant reduction in tic severity was only seen in the TS/OCD comorbid subgroup [79].

In children, an open-label pilot study tested the efficacy of low frequency rTMS targeting the SMA in ten males with TS ages 9–14 years. Over a 12-week period, the average resting motor threshold rose significantly, while tic severity decreased, although no significant effects on ADHD, anxiety, or depression were seen [80]. A similar study on the effects of daily rTMS targeting the SMA in children with TS also produced lower scores on the Yale Global Tic Severity Scale. A higher resting motor threshold post-treatment was found, in conjunction with lower scores on the Children’s Depression Inventory for depression and (Swanson, Nolan and Pelham) SNAP-IV inventory for ADHD [81]. The safety of TMS is documented by a review of 48 studies in children with various neurological conditions, which showed only minor side effects [82]. (Table 1).

## 7. Predictors of Transcranial Magnetic Stimulation Efficacy in Tourette Syndrome

An examination of predictors of efficacy of TMS for TS has resulted in preliminary considerations. In line with the mechanism of TMS therapeutics, the degree of brain electrical activity in individuals with TS might constitute a predictor variable. For example, ADHD has been associated with abnormal electrical activity (excess theta activity) [84], so that in those individuals with comorbid ADHD and TS, brain electrical abnormalities might be cumulative. In turn, TMS may be more efficacious in those subgroups of TS with greater electrical abnormalities (i.e., TS + ADHD). A study of deep TMS as an add-on treatment for intractable TS in adults showed no significant effect on tic severity overall in TS alone, but a significant decrease in comorbid TS [79]. Similarly, as described above, Chae et al. found that rTMS was more effective in comorbid TS compared to TS alone [78]. In both studies, TS was comorbid with OCD and ADHD (TS+OCD+ADHD), a complex phenotype that has shown to “breed true” as a TS subtype [6]. Overall, the few extant trials of TMS in TS suggest that the most improvement with TMS is shown in TS patients who are: younger, have comorbid ADHD/OCD and use SMA as a target (Table 1).

## 8. Future Directions

As discussed above, the comorbidity of TS with ADHD, and possibly OCD, plausibly confers additional abnormal cortical excitability compared with TS alone [67], which might account for the greater response to TMS treatment in “complex TS”, compared to those instances of TS without ADHD [78,79]. The hyperconnectivity between insula (an interoceptive center) and SMA (a motor planning center) observed in functional neuroimaging studies of TS further support the use of the SMA as a preferred target for TMS trials in TS. Future research in TMS trials in TS might additionally benefit from translational approaches measuring brain connectivity pre- and post-neuroimaging as well as neuroplasticity probes. Ultimately, the use of TMS in TS holds promise for an improved understanding of the pathophysiology and enhanced therapeutics in this debilitating condition.

## Figures and Tables

**Table 1:** Clinical Trials of Transcranial Magnetic in Children and Adults with Tourette Syndrome.

**Pediatric TS TMS Clinical Trials**									
**Authors**	**Design**	**Age (Range)**	**N**	**M:F**	**Comorbidities**	**Intervention**	**Comparison Groups**	**Targeted Brain Area**	**Results**
**Kwon, 2011 [80]**	Open label pilot study	9.57 ± 2.75 (9–14)	10	10:0	OCD (*n* = 1), ADHD (*n* = 3)	rTMS	None	SMA	Tic symptoms improved significantly, no improvement in ADHD, anxiety or depression.
**Le, 2013 [81]**	Open label	10.61 ± 2.18 (7–16)	25	22:3	Not specified	rTMS	None	SMA	Tic severity significantly lowered, also hyperactivity, attentional deficits, depression and anxiety lower with treatment.
**Adult TS TMS Clinical Trials**									
**Authors**	**Design**	**Age (range)**	**N**	**M:F**	**Comorbidities**	**Intervention**	**Comparison Groups**	**Targeted Brain Area**	**Results**
**Bloch, 2016 [79]**	Open label	32.6 ± 12.7 (20–61)	12	1:1	OCD (*n* = 6), ADHD (*n* = 4)	Deep rTMS	None	SMA	Tics did not improve overall but comorbid TS + OCD significant improvement tic severity.
**Chae, 2004 [78]**	Randomized, blinded crossover	34.9 ± 16.4 (13–60)	8	5:3	OCD (*n* = 4), ADHD (*n* = 3)	rTMS	None	L MC, L PFC	Tic and OCD symptoms improved significantly.
**Mantovani, 2006 [18]**	Open label	38.9 ± 11.9 (18–70)	10	8:2	OCD only (*n* = 5), OCD + TS (*n* = 2)	Low frequency rTMS	None	SMA	Tics and OCD symptoms significantly reduced up to 3 months.
**Munchau, 2002 [77]**	Single-blinded, placebo control	38.0 ± 13.2 (unknown)	16	3:1	OCD (*n* = 7)	rTMS	Sham/Placebo	L Premotor and L Motor Cortex	No significant improvement.
**Orth, 2005 [17]**	Single-blinded, placebo control	29.0 ± SD (19–52)	5	4:1	ADHD (*n* = 2)	rTMS	Sham	Left (and Bilateral) Premotor Cortex	rTMS no significant effect on global tic severity.
**Landeros-Weisenberger, A., 2015 [83]**	Randomized double-blind sham-control	33.7 ± 12.2 (unknown)	20	4:1	OCD (*n* = 5), ADHD (*n* = 8)	rTMS	Sham	SMA	No significant reduction of tic severity.

rTMS = repetitive transcranial magnetic stimulation; SMA = supplementary motor area; MC = Motor Cortex; PFC = Prefrontal Cortex; OCD; ADHD; PFC; SD.
